# Prospective comprehensive evaluation of an elastic-band beard cover for filtering facepiece respirators in healthcare workers

**DOI:** 10.1017/ice.2023.141

**Published:** 2024-01

**Authors:** Daryl Lindsay Williams, Benjamin Kave, Charles Bodas, Fiona Begg, Megan Roberts, Irene Ng

**Affiliations:** 1 Department of Anaesthesia and Pain Management, Royal Melbourne Hospital, Parkville, Victoria, Australia; 2 University of Melbourne, Parkville, Victoria, Australia; 3 Respiratory Protection Program, Royal Melbourne Hospital, Parkville, Victoria, Australia

## Abstract

**Objective:**

To undertake a healthcare-based multimodal evaluation of the combination of filtering facepiece respirator (FFR) with the elastic-band beard cover technique, including quantitative fit test (QNFT) results, skills assessment, and usability assessment.

**Design and setting:**

We conducted a prospective study through the Respiratory Protection Program at the Royal Melbourne Hospital from May 2022 to January 2023.

**Participants:**

Healthcare workers who required respiratory protection and could not shave for religious, cultural, or medical reasons.

**Intervention:**

Online education and personal face-to-face training on the use of FFR with the elastic-band beard cover technique.

**Results:**

Among 87 participants (median beard length 38 mm; interquartile range [IQR], 20–80), 86 (99%) passed 3 QNFTs consecutively with the elastic-band beard cover under a Trident P2 respirator and 68 (78%) passed 3 QNFTs consecutively with a 3M 1870+ Aura respirator. The first QNFT pass rate and the overall fit factors were significantly higher when using the technique than without the elastic-band beard cover. Most participants displayed a high skill level in their donning, doffing, and user seal-check techniques. Of 87 participants, 83 (95%) completed the usability assessment. The overall ease of use, comfort, and overall assessment were rated highly.

**Conclusions:**

The elastic-band beard cover technique can provide safe and effective respiratory protection for bearded healthcare workers. The technique was easily taught, comfortable, well tolerated and accepted by healthcare workers, potentially allowing them full participation in the workforce during pandemics with airborne transmission. We recommend further research and evaluation of this technique in a broader health workforce.

Airborne contaminants can range from several micrometers to fractions of a micrometer, whereas human hair has an average thickness of ∼100 µm.^1^ Facial hair, even as short as 1 mm in length, in the seal zone of tight-fitting respirators, is known to decrease respiratory protection.^2–6^ Employers have an obligation to keep their staff safe under occupational health and safety regulations, and staff have an obligation to wear their respirators consistent with manufacturers and jurisdictional recommendations.^7–10^ International standards agencies recommend that individuals who have stubble, a moustache, sideburns, or a beard that passes between the skin and the sealing surface should not wear a tight-fitting respirator, whether full or half facepiece.^9–11^ The requirement of staff to be clean shaven when wearing a filtering facepiece respirator (FFR) may be considered indirect discrimination against those who are unable to shave for medical, cultural, or religious reasons, even though it is likely to be a genuine and lawful request by employers.^12^

An undermask elastic-band beard cover, also known as the Singh Thattha technique,^6^ has been identified as a potential option for those who are unable to shave in the healthcare industry, in which both respiratory protection and source control is required. This technique involves the use of a long elastic-band stretched tightly over the beard, with the band acting as an artificial skin, forming a seal with the respirator. The original study by Singh et al,^13^ included a small cohort of 27 participants, who predominantly underwent qualitative fit-testing only. Subsequently, another study was published, demonstrating an acceptable quantitative fit test (QNFT) pass rate with at least 1 type of FFR in 30 participants.^14^ Various numbers and types of FFRs were tested for each participant in this study, and participants received brief and nonstandardized training on the technique. Unfortunately, the study lacked clinical evaluation and 40% of the participants were not healthcare workers.

Health authorities and occupational health organizations do not currently recommend the adoption of the Singh Thattha technique, due to limited evidence in published studies.^15^ To address this research gap, we studied the elastic-band beard cover technique, using a multi-modality evaluation on healthcare workers, based on the “Project BREATHE” framework for respiratory protective equipment (RPE), as recommended by the Centers for Disease Control and Prevention and the National Institute for Occupational Safety and Health.^16^ The assessment included QNFTs, skills assessment, usability assessment, and a plan for repeat assessments at prespecified intervals. We sought to determine whether the combination of FFR and elastic band can provide safe and effective respiratory protection for bearded healthcare workers, whether it interferes with occupational activities, and whether it is comfortable and tolerable for the duration of wear.

## Methods

The full study methodology has been published.^17^ In brief, this prospective study was conducted through the Respiratory Protection Program at the Royal Melbourne Hospital from May 2022 to January 2023. This study was approved by Melbourne Health Human Research Ethics Committee (no. QA2022022). Both internal and external healthcare workers were invited to participate if they required respiratory protection and could not shave for religious, cultural, or medical reasons.

Interested participants completed an online survey that gathered information regarding demographics, work hazards, training, and experience with FFR and attitude. An online education package on the use of RPE and the elastic-band beard cover, TheraBand (TheraBand, Akron, OH) for FFR technique was provided. This was followed by a face-to-face session, where the participant received one-on-one training; underwent a skills assessment on their donning, doffing and user seal check techniques; completed QNFTs and a usability and comfort survey. A standard operating procedure was followed throughout the session.

We used 2 types of 3-panel flat-fold N95/P2 FFRs for this study: 3M 1870+ Aura (3M, St. Paul, MN, USA) and Trident P2 respirator (Industree, West Gosford, NSW, Australia). These 2 types of FFRs were chosen because they were the most readily available N95/P2 FFRs in our jurisdiction.^18^ They have been shown to have very high QNFT pass rates and high comfort and usability ratings in healthcare workers.^19^

A baseline QNFT assessment without the TheraBand (naked beard) was completed first, followed by 3 consecutive QFNTs wearing the TheraBand, for each make of the 2 FFRs. The participant was required to repeat the donning and doffing procedures (including the TheraBand) for each of the 3 QNFTs. The skills assessment was performed by a trained fit tester using a standardized marking system, of which the interrater reliability was also assessed.^17^ The participant was then required to complete an online usability and comfort survey after the face-to-face session.

We sought to determine whether the elastic-band beard cover technique could reliably provide adequate respiratory protection. Therefore, the primary outcome was to investigate the percentage of participants, with the TheraBand on, who could achieve 3 consecutive QNFT passes (overall fit factor >100 for each of the 3 tests) with either of the 2 types of FFRs tested. Secondary outcomes included (1) the percentage of participants who achieved a first-time QNFT pass wearing the TheraBand; (2) a comparison of first-time QNFT pass rates and fit factors between the elastic-band beard cover technique and the naked beard; (3) skills assessment results; and (4) usability and comfort survey results. We also investigated whether there was any association between beard length and overall fit factor. We planned to repeat the QNFT and the skills and user assessments for all participants at 3 and 12 months. However, here we present only the findings from the initial assessment.

### Statistical analysis

Descriptive statistics have been used to present the demographic data, QNFT results, skills assessment results, and usability and comfort assessment results. The McNemar test was used to compare the QNFT pass rates, and the Wilcoxon signed-rank test was used to compare the fit factors between TheraBand elastic-band beard cover technique and the naked beard. Pearson correlation was used to analyze the association between beard length and overall fit factor. The κ statistic was used to assess interrater reliability in the skills assessment. Statistical analyses were performed using Stata version 13.0 software (Statacorp, College Station, TX, USA).

Because the primary outcome was to examine the percentage of participants who could pass 3 consecutive QNFTs with the elastic-band beard cover technique for both types of FFRs and we did not compare the 2 types of FFRs, we did not calculate sample size for this study. We acquired the largest possible convenience sample over a 9-month period; therefore, the sample size was dependent on the number of suitable candidates from various statewide healthcare organizations.

## Results

**Figure 1.**
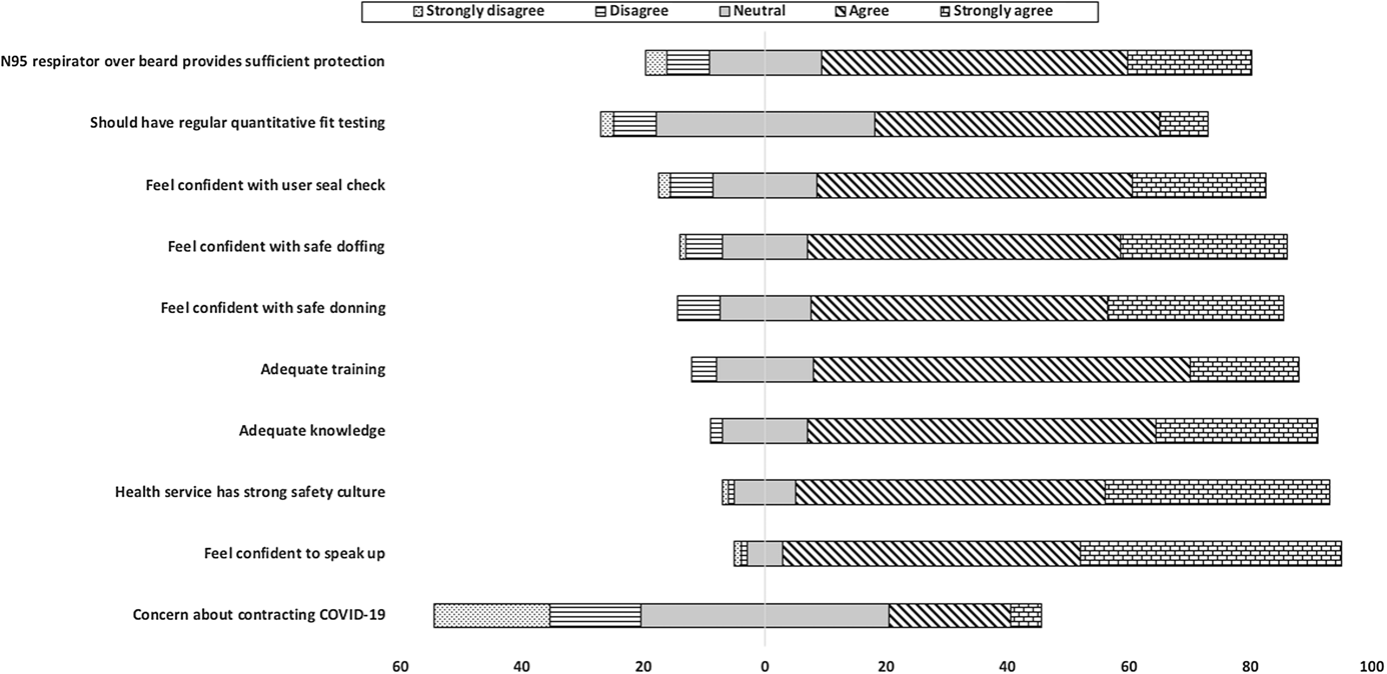
Attitude survey before training and quantitative fit testing.

In total, 87 male healthcare workers participated in this study. For this cohort, data regarding demographic information, work hazard assessment, health safety screen, and training and experiences with N95/P2 respirators are presented in Table 1. The median beard length was 38 mm (IQR, 20–80; range, 5–750). Of the 87 participants, 84 (97%) were not able to shave for religious or cultural reasons. Half of the participants had undertaken alternative duties due to lack of beard-compatible RPE. Also, 21 participants (24%) were working in aerosol-generating environments, and 70 (80%) of the participants had worn RPE over their naked beard to protect themselves from SARS-CoV-2. Moreover, 49 participants (56%) had received formal education and training on RPE. Figure 1 shows the participant attitudes toward RPE before the study commenced.

**Table 1.** Participant Demographics, Work Hazard Assessment, Medical and Health Safety Screen, and Training and Experiences With N95/P2 Respirators^a^

Variable	Total (N = 87), No. (%)^a^
Age, yrs	31.9 +/− 11.2
Sex, M:F	87:00:00
BMI, kgm^-2^	26.2 +/− 4.7
Role	
*Student*	32 (37%)
*Nursing*	24 (28%)
*Medical*	5 (5.5%)
*Paramedics*	5 (5.5%)
*Clinical assistant*	7 (8%)
*Administrative*	3 (3.5%)
*Security*	4 (4.5%)
*Other*	7 (8%)
Years of healthcare experience	3 (1-9[0-29])
Reason for not being able to shave:	
*Cultural/religious*	84 (97%)
*Medical*	2 (2%)
*Other*	1 (1%)
Beard length, mm	38 (20-80[5-750])
Required to undertake alternate duties due to lack of suitable respiratory protective equipment for bearded workers over the past 3-6 months:	
*All the time*	11 (13%)
*Most of the time*	11 (13%)
*Some of the time*	15 (17%)
*Rarely*	6 (7%)
*Never*	44 (50%)
Have been wearing respiratory protective equipment over the beard to protect oneself from SARS CoV-2 over the past 3-6 months:	
*All the time*	42 (48%)
*Most of the time*	17 (20%)
*Some of the time*	8 (9%)
*Rarely*	3 (3%)
*Never*	17 (20%)
Number of N95/P2 respirators used over the last 4 weeks while working in current role:	
*0*	18 (21%)
*10-Jan*	22 (25%)
*20-Nov*	14 (16%)
*21-30*	14 (16%)
*>30*	14 (16%)
*Missing data*	5 (6%)
Number of N95/P2 respirators used over the last 3 months while working in current role:	
*0*	15 (17%)
*20-Jan*	16 (18%)
*21-40*	5 (6%)
*41-60*	14 (16%)
*>60*	32 (37%)
*Missing data*	5 (6%)
Work hazard assessment	
1. When working in respiratory protective equipment (RPE), participants are involved in:	
a. Temperature exposure > 30°C for >15 mins	13 (15%)
b. Heavy cleaning for >30 mins at a time	3 (3%)
c. Lifting heavy loads (>20kg for >2 mins) several times per shift	8 (9%)
d. Fast-paced walking for >10 mins several times per shift	18 (21%)
e. None of the above	62 (71%)
2. Involved in aerosol-generating (AG) procedures or in contact with patients with AG behaviours	21 (24%)
Medical/health safety screen	
1. Claustrophobia limiting ability to wear a face mask	2 (2%)
2. Lung, heart, or other problems limiting ordinary physical activity or impairing ability to perform usual roles at work	1 (1%)
3. Work limiting symptoms when performing usual tasks at work	1 (1%)
4. Wear corrective lenses at work	1 (1%)
5. Type of corrective lenses	24 (28%)
*Glasses*	21 (24%)
*Contact lenses*	3 (3%)
Training and experiences with N95/P2 respirators	
1. Received formal education and training on RPE in past 12 months	49 (56%)
2. If so, how long ago (months)	2 (0-9[0-12])
3. Format of education delivered	2 (0-9[0-12])
*Information brochure*	6 (7%)
*Face-to-face learning*	36 (41%)
*Online education*	26 (30%)
*Other*	0 (0%)
4. Received formal assessment on ability to safely don/doff and complete fit check of N95 respirators	35 (40%)
5. Who performed the assessment	
*Infection prevention surveillance team*	7 (8%)
*Working area/department*	7 (8%)
*Clinical educators*	16 (18%)
*Other*	10 (11%)

Note. SD, standard deviation.

a Units unless otherwise specified.

Of the 87 participants, 86 (99%) were able to pass 3 QNFTs consecutively with the elastic-band beard cover under a Trident P2 respirator. For the 3M 1870+ Aura respirator, 68 (78%) were able to achieve 3 consecutive QNFT passes (Table 2). The first QNFT pass rate and the overall fit factors were significantly higher (*P* < .001) for both types of FFRs when the elastic-band beard cover technique was used, compared to the naked beard (Table 2). When the elastic-band beard cover was worn, the first QNFT pass rates were 99% for the Trident P2 respirator and 91% for the 3M 1870+ Aura respirator. With the naked beard, these rates were 44% and 17%, respectively (*P* < .001).

**Table 2.** Quantitative Fit Test (QNFT) Results of Trident P2 Respirator and 3M 1870+ Aura Respirator With and Without the Elastic-band Beard Cover Technique^a^

Variable	Without Elastic-Band Beard Cover (n = 87), No. (%)^a^	With Elastic-Band Beard Cover (n = 87), No. (%)^a^	*P* Value
**Trident P2 respirator**			
Passed three consecutive QNFTs	N/A	86 (99%)	<0.001
Passed the first QNFT	38 (44%)	86 (99%)	<0.001
Overall fit factor	81 (25-201[4-201])	201 (198-201[43-201])	N/A
**3M 1870+ Aura respirator**			
Passed three consecutive QNFTs	N/A	68 (78%)	p < 0.001
Passed the first QNFT	15 (17%)	79 (91%)	p < 0.001
Overall fit factor	19 (12-72[2-201])	170 (127-201(8-201)]	N/A

Note. N/A, not applicable; IQR, interquartile range.

a Units unless otherwise specified.

Most participants displayed a very high skill level in their donning, doffing, and user seal-check techniques when using the elastic-band beard cover with the FFR. The median total score in their skills assessment was 33 (IQR, 31–33), which was the highest possible score. Table 3 lists the details of the skills assessment results. There was good interrater reliability (κ coefficient, 0.95 between 2 observers).

**Table 3.** Skills Assessment Results

Variable	Score, Median (IQR; range)
Preparation (maximum score, 6)	6 (6–6; 4–6)
Donning (maximum score, 11)	11 (11–11; 10–11)
User seal check (maximum score, 3)	3 (3–3; 0–3)
Doffing (maximum score, 13)	13 (12–13; 9–13)
Total score (maximum score, 33)	33 (31–33; 26–33)

Note. IQR, interquartile range.

Of 87 total participants, 83 completed the usability and comfort survey, for a response rate of 95%. Moreover, 12 participants had used the elastic-band beard cover technique in clinical setting at the time of survey completion. Also, 99% agreed or strongly agreed that there was adequate education and training on the use of the TheraBand and that they could repeatedly complete safe donning procedures (Table 4). All participants felt that they could safely doff the TheraBand. While using the TheraBand and respirator, >80% felt that they could hear adequately; 83% felt that they could speak clearly; and 86% felt that they were understood.

**Table 4.** Usability and Comfort Assessment^a^

Variable	Total (N = 83), No. (%)
Preferred model of respirator to wear with a Theraband^®^	
*3M Aura 1870+*	53 (64%)
*Trident*	30 (36%)
Feel that education and training was adequate regarding the use of the Theraband^®^	
*Agree or strongly agree*	82 (99%)
**Usability assessment**	
Comfortable with the preparation and inspection of the Theraband^®^	
*Agree or strongly agree*	82 (99%)
Can repeatedly safely don the Theraband^®^ and respirator	
*Agree or strongly agree*	82 (99%)
Pass user seal check when wearing the Theraband^®^ under the respirator	
*Every single time*	57 (69%)
*Most of the time*	23 (28%)
*Sometimes*	2 (2%)
*Rarely*	0 (0%)
*Never*	1 (1%)
Can safely doff the Theraband^®^	
*Agree or strongly agree*	83 (100%)
Able to hear adequately while using the Theraband^®^ and respirator	
*Agree or strongly agree*	77 (83%)
Able to speak clearly and be understood while using the Theraband^®^ and respirator	
*Agree or strongly agree*	71 (86%)
Theraband^®^ is not interfering with the rest of the personal protective equipment	
*Agree or strongly agree*	83 (100%)
Find the Theraband^®^ and respirator provides a good seal	
*Agree or strongly agree*	82 (99%)
Feel well protected from respiratory hazards while wearing the Theraband^®^ and respirator	
*Agree or strongly agree*	82 (99%)
Work tasks affected due to the use of the Theraband^®^ and respirator combination	
*Using stethoscope, yes*	11 (13%)
*Wearing spectacles/glasses, yes*	14 (17%)
*Other task, yes (talking on the phone)*	1 (1%)
**Comfort assessment**	
Firmness of the fit of the Theraband^®^ on the face	
*Too tight*	7 (9%)
*About right*	74 (90%)
*Too loose*	1 (1%)
Breathability of the respirator when using the Theraband^®^	
*Poor*	2 (2.5%)
*Average*	25 (30%)
*Good*	56 (67.5%)
Do not get too hot when wearing the Theraband^®^ and respirator	
*Agree or strongly agree*	66 (80%)
Do not perspire excessively when wearing the Theraband^®^ and respirator	
*Agree or strongly agree*	61 (73%)
Discomfort	
*Eye periorbital irritation, yes*	0 (0%)
*Skin irritation or rash, yes*	1 (3%)
*Anxiety limiting use, yes*	2 (2%)
Pressure area	
*Nose, yes*	8 (10%)
*Cheeks, yes*	6 (7%)
*Ears, yes*	5 (6%)
*Chin, yes*	9 (11%)
*Other, yes (upper jaw)*	1 (1%)

a Values are expressed as mean ±SD or number (%).

**Figure 2.**
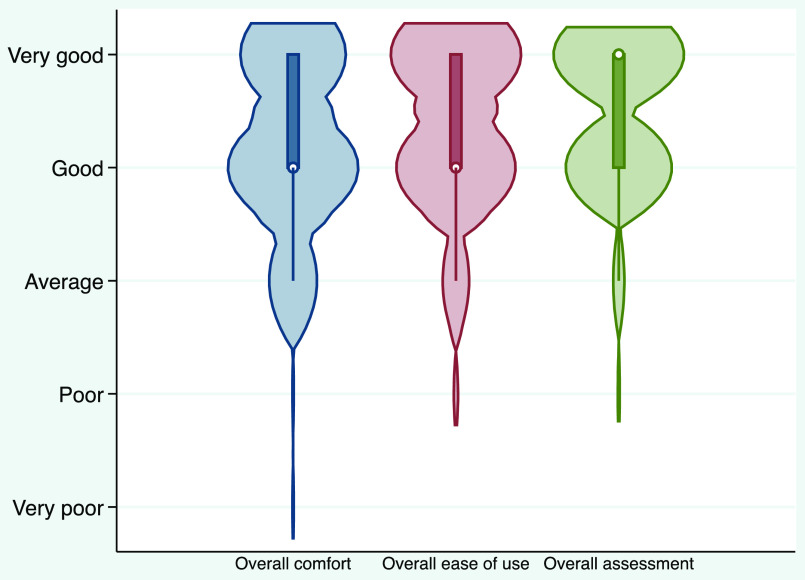
Violin plot to show overall comfort, overall ease of use, and overall assessment of the elastic-band beard cover technique.

Regarding the comfort assessment of the elastic-band beard cover technique, 90% of participants felt that the firmness on the face was “about right,” and 67.5% felt that the breathability was good (Table 4). Overall, 80% agreed or strongly agreed that they did not get too hot, and 73% felt that they did not perspire excessively. The overall ease of use, overall comfort, and overall assessment were rated highly, as shown in the violin plot in Figure 2.

**Figure 3.**
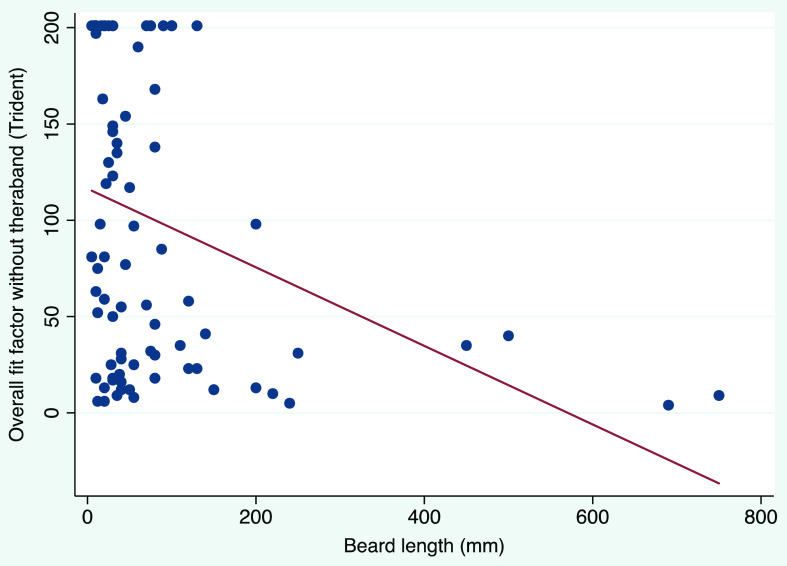
Scatter plot and fitted line to show the correlation between beard length and overall fit factors for participants who wore the Trident P2 respirator without the elastic-band beard cover. Note. r = −0.34 and *P* = .001.

We detected a weak association between beard length and overall fit factor when the participants undertook QNFTs without the elastic-band beard cover: r = −0.34 for the Trident P2 respirator and r = −0.29 for 3M 1870+ Aura respirator (Fig. 3).

## Discussion

Tight-fitting respirator face masks, such as FFRs, are considered the reference standard RPE for healthcare workers who may be exposed to aerosol-transmitted diseases.^3,12^ This study represents the first description of a multimodal evaluation of the elastic-band beard cover technique in a broad range of healthcare workers, using a peer-reviewed standardized protocol for education, training, and assessment.^17^

Our findings of very high first and consecutive QNFT pass rates, especially with the Trident P2 respirator, indicated that the elastic-band beard cover technique, when correctly taught and practiced, was a practical and effective method of achieving reliable and efficacious respiratory protection in this population of bearded healthcare workers. This finding is significant for bearded healthcare workers who have faced a unique challenge during the COVID-19 pandemic when they are required to use FFRs and for those who have, in numerous instances, been excluded from clinical work due to their inability to participate in standard respiratory protection programs.^20^ Several benefits can potentially be gained through increased adoption of this protocol for bearded healthcare workers.

The most significant benefit of our findings is improved safety. We have demonstrated that the use of the elastic-band beard cover can significantly improve the QNFT pass rate and fit factor for bearded healthcare workers wearing FFRs. This information has the potential to improve the available pool of healthcare workers and to prevent the removal of staff from vital roles at times of peak pandemic pressure. By providing a solution that allows bearded healthcare workers to use RPE effectively, the health industry can ensure that individuals are not excluded based on cultural or religious beliefs, or physical appearance, which was a point of contention during the pandemic. Published legal opinions have determined that an employer likely can require employees to be clean shaven for their health and safety, but it is preferable to avoid any appearance of discrimination by considering alternatives to redeployment,^20^ and this study confirms the existence of a reasonable alternative.

Until now, conventional thinking in the science of respiratory protection has been that the Singh Thatta technique is not an acceptable form of respiratory protection. This perception has been perpetuated by the paucity of published research into the technique, its contravention of international standards that mandate a clear interface between mask and skin, and the fact that the technique contravenes mask manufacturer instructions.^15^ Hopefully, our findings will prompt consideration of the technique and further research regarding broader participation of bearded employees in the health workforce.

Another potential benefit of this study is to increase health workforce awareness around the issue of facial hair and its impact on respiratory protection. Our study was conducted several years into the COVID-19 pandemic, and our results demonstrated that 50% of the participants had never had to move to alternative duties, even though 56% of the participants had received training on using FFRs and 48% had been wearing FFRs “all the time” over their beard at work over the 3–6 months preceding their involvement in the trial. Given these data, one can deduce that health industries in many instances did not enforce clean-shaven practice with FFRs and allowed bearded healthcare workers to continue their duties without appropriate RPE. This situation was potentially due to the difficulty of enforcing the recommendations. Furthermore, other reported studies have indicated that many bearded study participants were not aware that their safety was potentially jeopardized.^5,21^ Our findings should empower both health administrators and staff to acknowledge that a viable solution for respiratory protection in this cohort of workers exists and that the education and training pathway is implementable.

Significantly, the technique was well accepted by our participants, as demonstrated by the usability and comfort assessment. The high rating scores for overall comfort and ease of use indicated that the elastic-band beard cover technique was practical and well tolerated. One important aspect that needs to be explored and improved upon is the interference with equipment, such as eyeglasses and stethoscopes, due to TheraBand placement over the ears. Also, some infrequent responses to the survey signaled discomfort related to perspiration and heat when using the technique. Further studies are required to investigate the thermal microenvironment inside the respirator with the elastic-band beard cover technique.

This study had several limitations. First, the technique was taught in a controlled environment with skilled respiratory protection program technicians. Rolling out the technique to a broader population may dilute the quality of training and therefore results. Second, our study participants were volunteers who were highly motivated to work towards a solution that would confer adequate respiratory protection in the presence of their beards. Results may differ when applied to a broader population. Third, we used 2 types of FFRs, both of which had reportedly very high QNFT pass rates compared to other FFR types. The technique would require further validation if FFRs with lower QNFT pass rates were utilized.

In conclusion, our study shows that the elastic-band beard cover technique can provide safe and effective respiratory protection for bearded healthcare workers. With a standardized education and training approach, it can provide consistent efficacy. The skills of the technique can easily be taught and mastered by healthcare workers. The technique is relatively comfortable, well tolerated, and well accepted by healthcare workers, allowing them an avenue to full participation in the workforce during pandemics with airborne transmission. We recommend further research and evaluation of this technique as it is adopted by the broader health workforce.
